# Quality of Life of Informal Caregivers behind the Scene of the COVID-19 Epidemic in Serbia

**DOI:** 10.3390/medicina56120647

**Published:** 2020-11-26

**Authors:** Natasa Todorovic, Milutin Vracevic, Nina Rajovic, Vedrana Pavlovic, Petar Madzarevic, Jelena Cumic, Tanja Mostic, Nikola Milic, Tatjana Rajovic, Rosa Sapic, Petar Milcanovic, Igor Velickovic, Slobodan Culafic, Dejana Stanisavljevic, Natasa Milic

**Affiliations:** 1 Red Cross of Serbia, 11000 Belgrade, Serbia; natasa@redcross.org.rs (N.T.); milutin@redcross.org.rs (M.V.); 2 Institute for Medical Statistics and Informatics, Faculty of Medicine, University of Belgrade, 11000 Belgrade, Serbia; nina.rajovic@med.bg.ac.rs (N.R.); vedrana.pavlovic@med.bg.ac.rs (V.P.); petar.madzarevic@gmail.com (P.M.); nmilic1996@gmail.com (N.M.); tanja.rajovic63@gmail.com (T.R.); petar.milcanovic@gmail.com (P.M.); dejana.stanisavljevic@med.bg.ac.rs (D.S.); 3 Department of Anesthesiology, Clinical Center of Serbia, 11000 Belgrade, Serbia; jelena.cumic@gmail.com (J.C.); tanja.mostic@gmail.com (T.M.); 4 Department of Occupational Therapy, College for Social Work, 11000 Belgrade, Serbia; sapicdr@gmail.com; 5 Medical School, Academy of Vocational Studies Belgrade, 11000 Belgrade, Serbia; velickovicigor@live.com; 6 Department of Interventional Neuroradiology, Special Hospital for Cerebrovascular Diseases “Saint Sava”, 11000 Belgrade, Serbia; sanjauma@gmail.com; 7 Department of Internal Medicine, Division of Nephrology and Hypertension, Mayo Clinic, Rochester, MN 11000, USA

**Keywords:** informal caregivers, COVID-19, quality of life, mental health

## Abstract

*Background and objectives*: The COVID-19 pandemic has had an unprecedented reliance on informal caregivers as one of the pillars of healthcare systems. The aim of this study was to assess the quality of life of informal caregivers during the COVID-19 epidemic in Serbia. *Materials and Methods*: A cross-sectional study was conducted among informal caregivers during the COVID-19 epidemic in Serbia. Physical and mental quality of life was measured by the 36-Item Short-Form Health Survey. Additional data included sociodemographic characteristics, caregiver and care recipient characteristics, and COVID-19 related concerns. The qualitative component was performed using focus groups and individual in-depth interviews. *Results*: Out of 112 informal caregivers enrolled, most were female (80%), and the average age was 51.1 ± 12.3 years. The majority was delivering care to one person, who was a family member, on a daily basis (86.4%, 92%, and 91.1%, respectively). In multiple regression models, significant predictors of caregivers’ physical health were delivering care to a family member and a higher level of care complexity, while significant predictors of caregivers’ mental health were a higher level of care complexity and increased concerns about self-health and the health of the person being cared for due to the COVID-19 epidemic. *Conclusions*: Informal caregivers are experiencing negative physical and mental health outcomes during the COVID-19 epidemic in Serbia.

## 1. Introduction

The COVID-19 epidemic has placed pressure on various national healthcare systems worldwide [1]. It has disrupted society on a global scale and exacerbated feelings of fear, anxiety, and isolation [2]. Due to accelerated growth in caseloads and resource constraints in health systems, the focus was placed on early screening and hospitalization of severe cases while leaving the need for homecare overlooked. In such pandemic emergencies, homecare is the only option for people without healthcare facility access, with financial issues, and for people living in resource-constrained and low-income settings. This steered an unprecedented reliance on caregivers as one of the pillars of healthcare systems. The role of the caregiver has become more important in today’s society, shouldering a significant responsibility in healthcare delivery, local communities decision making, care for families and individuals, but also in delivering social protection and care on a long-term basis [3].

Informal caregivers are individuals who deliver care to children and adults with disabilities, mental disorders, those who are chronically ill, as well as older family members and friends with specific needs, who may live within or outside the caregiver’s home [4,5]. According to the Organization for Economic Cooperation and Development (OECD) [6], 13% of individuals older than 50 years deliver informal care to at least one person weekly. However, there is a strong difference in the specific requirements of people receiving care. Most caregivers perform a wide spectrum of activities, such as personal hygiene, assuring patient compliance, and/or supply [5,7]. The delivery of these forms of care and personal assistance can be both demanding and time-consuming and can often lead to physical and psychological burdens for the caregiver [5,8].

Previous research has shown that 10% of informal caregivers experience a decline in physical health over the course of healthcare delivery [9,10]. Caregivers have been known to develop chronic diseases, such as cardiovascular disease, diabetes, arthritis, and malignancies, at almost double the rate compared to those who are not a caregiver [11]. Multiple studies have shown that poor mental health is widespread in caregivers, with depression and anxiety being the most common symptoms [12,13]. Extended periods of physical and psychological efforts, associated with high-level uncertainty and inability to adequately balance between work, private life, and providing care, have a negative impact on the emotional and psychological well-being of the informal caregivers.

The COVID-19 pandemic has dealt a heavy blow to the caregivers in regards to the circumstances they face daily. In a short time, informal caregivers became aware of the increased risk of COVID-19 for those they care for, as well as for themselves. In the pandemic, informal caregivers have been working without proper training, protocols, adequate medical equipment, organizational support, and other resources that are to some extent available to formally paid caregivers working within institutionalized programs. Informal caregivers who deliver home care during public health emergencies, such as COVID-19, are a valuable human resource that increases the healthcare capacities of society in general, but also more specifically in aging populations and regions with suboptimal healthcare systems. However, informal caregivers are facing significant challenges during the COVID-19 pandemic, and yet, our knowledge about the impact of healthcare delivery on their physical and mental well-being is limited.

In this study, we aimed to examine the impact of informal care delivery during the COVID-19 epidemic in Serbia on caregivers’ physical and mental quality of life. This would serve as a solid ground to refine the public health policies aiming to ease the healthcare delivery burden on informal caregivers during the COVID-19 pandemic.

## 2. Methods

This was a cross-sectional study conducted among informal caregivers from March to May 2020, during the COVID-19 epidemic in Serbia. The study was performed in collaboration with the Red Cross of Serbia, the humanitarian organization Caritas Serbia, and the Faculty of Medicine, University of Belgrade. Quantitative and qualitative components were included. Participation was voluntary. Ethical approval was obtained from the Institutional Review Board (IRB) of the Faculty of Medicine University of Belgrade (Ethical code: 16/2020, approved date: 23 March 2020). The purpose of the study was explained to the participants, and oral consent was obtained and documented in the records at the beginning of the study. The IRB approved the use of oral consent, as there was no potential harm to the study participants.

### 2.1. Quantitative Component

Quality of life was measured by the 36-Item Short-Form Health Survey (SF-36) (Serbian version). The SF-36 consists of 36 questions, classified into eight domains: physical function, physical role, body pain, general health, vitality, social functioning, emotional role, and mental health, ranging from 0 to 100, with higher values indicating a better quality of life. The quality of life domains were summarized into two dimensions of quality of life: physical health and mental health. Additional data included sociodemographic characteristics of the caregiver, the frequency and duration of informal care, number of care recipients, and details regarding family membership. The level of care complexity was assessed on a scale, ranged from 0–10, where 0 represented “not difficult at all” and 10 “extremely difficult”. Two questions related to the impact of the COVID-19 pandemic on the caregiver’s health were posed. In order to define recommendations regarding caregiver support during the COVID-19 epidemic, an assessment of caregiver needs was done. The questionnaire was distributed via social networks and Sowa Media and Penzin web-based portals, dedicated to quick and reliable information share regarding current societal problems, culture, and human rights, as well as elderly problems and intergenerational cooperation, respectively.

### 2.2. Qualitative Component

The qualitative component was performed using focus groups and individual in-depth interviews. All participants gave informed consent to participate in the research, as well as permission to be recorded. The study participants were allowed to withdraw consent at any time.

Interview outline: The interview outline was determined by seeking expert opinion and consulting the relevant literature. The main focus groups and interview questions posed to participants were related to informal caregivers’ health self-assessment and COVID-19 current needs. Data collection and analysis: The purpose of the study was communicated with the participants in advance, and an interview time was scheduled at their convenience. The interviewer possessed a Master of Psychology and had more than 30 years of experience in qualitative research and humanitarian work among the elderly and people with disabilities. Interviews were managed according to a previously defined protocol. Since the research was conducted during the COVID-19 pandemic, focus groups were realized via the video-conference Zoom application, while interviews were done in a one-to-one manner by telephone. The interviews and focus groups took 60 to 90 min. The interviewer established good relationships with the participants but stayed neutral when collecting data. Active listening with unconditional acceptance and clarification were used to promote the authenticity of the data and avoid bias. Conversations were recorded, and notes were kept. The answers were summarized according to key questions and transcribed using the original answer format (as they were pronounced). The answers were grouped by categories, and the ones with the highest frequency were selected to define the most fundamental problems.

### 2.3. Statistical Analysis

Numerical data were presented as mean with standard deviation or with median with ranges. Categorical variables were summarized by absolute numbers with percentages. Differences in physical and mental health among informal caregivers according to sociodemographic factors, caregiver and care recipient characteristics, and COVID-19 related questions were analyzed by the Students’ test for independent samples. Correlations between numerical variables and quality of life domains were assessed by Pearson’s correlation coefficients. Linear regression models were used to assess predictors, such as sociodemographic characteristics, caregiver and care recipient characteristics, and COVID-19 related concerns for caregiver’s physical and mental health (as dependent variables). In all analyses, the significance level was set at 0.05. Statistical analysis was performed using IBM SPSS statistical software (SPSS for Windows, release 25.0, SPSS, Chicago, IL, USA).

## 3. Results

### 3.1. Quantitative Research

**Figure 1:**
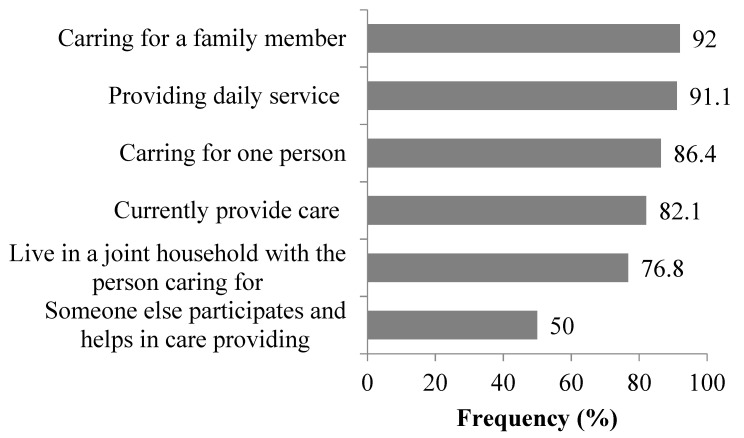
Distribution of respondents in relation to the characteristics of care. Bars represent the percentage of the caregiver’s preference for each statement.

Informal caregivers were enrolled (*n* = 112). Most caregivers were female (80%), and the average age was 51.1 ± 12.3 years (Table 1). Most were living in urban areas (80%), and half were employed (47%). The majority were delivering care to one person, a family member, on a daily basis (86.4%, 92%, and 91.1%, respectively). Most were living in a joint household with the person they care for (76.8%) (Figure 1). The median time spent delivering care was five years, ranging from several months to 50 years. The average level of care complexity was 7.2 ± 2.5, measured on a scale of 0 (not at all difficult) to 10 (extremely difficult). The reported average Physical and Mental health dimension scores were 54.1 ± 21.7 and 49.7 ± 22.5, respectively. Informal caregiver’s quality of life, according to sociodemographic characteristics, is presented in Table 2. Informal caregivers who were retired had lower scores on the physical health dimension compared to other categories of employment status (*p* = 0.030).

Informal caregivers that provide healthcare to family members had lower physical and mental health dimension scores in contrast to those providing care for care recipients who were not family members (*p* = 0.001 and *p* = 0.002, respectively) (Table 2). There was a negative correlation between the level of care complexity and the physical and mental quality of life of informal caregivers (*p* = 0.001 and *p* < 0.001, respectively) (Table 2).

During the epidemic, 65% of informal caregivers stated that they had increased concerns about self-health and the health of the person being cared for due to the COVID-19 epidemic, while 40% believed that their health was more endangered compared to the period before the epidemic. Those having increased concerns about self-health and the health of the person being cared for, and those believing that their health was more endangered during the pandemic, reported lower physical health dimension scores of quality of life (*p* = 0.022 and *p* = 0.048, respectively) (Table 2).

In the univariate regression analysis, the significant predictors of physical health were: retired status (*p* = 0.030), providing care for family member (*p* = 0.001), level of care complexity (*p* = 0.001), belief that caregiver health was more endangered compared to the period before the epidemic (*p* = 0.048), and increased concerns about self-health and the health of the person being cared for due to the COVID-19 epidemic (*p* = 0.022). Providing care for a family member and level of care complexity were predictors of the respondents’ physical health in multivariate analysis (Table 3).

In the univariate regression analysis, the significant predictors of mental health were: providing care for a family member (*p* = 0.002), the level of care complexity (*p* < 0.001), and increased concerns about self-health and the health of the person being cared for due to the COVID-19 epidemic (*p* = 0.006). The level of care complexity and fear for oneself and increased concerns about self-health and the health of the person being cared for due to the COVID-19 epidemic were predictors of respondents’ physical health in the multivariate analysis (Table 3).

**Figure 2:**
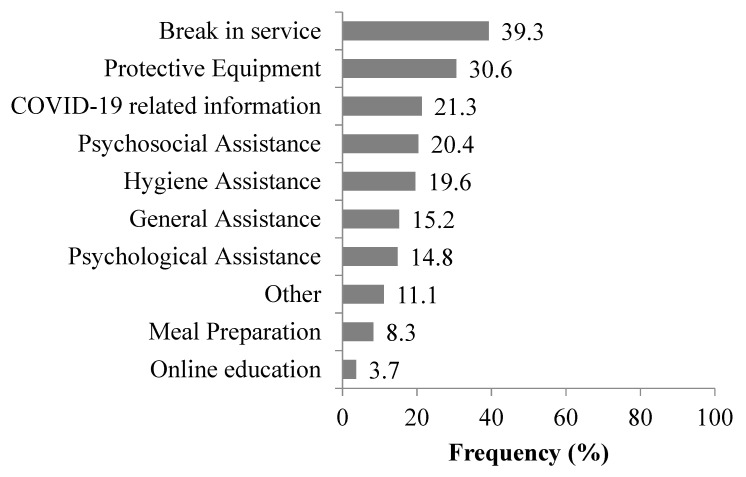
Most frequent needs of informal caregivers during the COVID-19 epidemic in Serbia. Each bar represents the percentage of the caregiver’s need.

The most frequent needs informal caregivers have reported were: break in service (39.3%), personal protective equipment (30.6%), and COVID-19 related information (21.3%). Other reported needs of informal caregivers during the COVID-19 epidemic in Serbia are presented in Figure 2.

### 3.2. Qualitative Research

Within the scope of qualitative research, two focus groups and six in-depth interviews were performed with a total of 18 participants, all informal caregivers, of which 16 were women and two men. Some of the most representative statements were as follows: “It is hard, sometimes I get up like a train ran over me, I get up more tired than that I went to bed” (59-year-old woman); “It is important that my son feels good, if he is well, I am also well. I do not think about my health when I think about him” (74-year-old man); “I sleep a little for years, I take care of my mother-in-law for 6 h, but I also do hard physical work on the property. It is very difficult for me, I no longer have my life, I spent half of my married life in care, I cared more for her than for my children”(45-year-old woman); “Life is as it should be, I take medicine in the evening, calm down, he sleeps at night, so I can get some rest” (woman 62 years old); “Why were gerontocarer services cancelled during the epidemic? Corona did not reduce our needs for gerontocarer support, on the contrary, it increased it” (69-year-old man); “In the epidemic my greatest fear is that my appendix may rupture or that I may need urgent gallstone surgery. Would they admit me into a hospital or is it all just COVID now?” (82-year-old woman); “Now, in the epidemic I am more afraid me or my husband’s health will take a turn for the worse. And if I fall ill, who will take care of him?” (51-year-old woman).

The obtained results from quantitative and qualitative research served to define recommendations for improving the physical and mental health of informal caregivers during the COVID-19 epidemic in Serbia.

## 4. Discussion

Caregiving is known to have an influence on informal caregivers’ quality of life, yet little is known about the added burden of providing care during the COVID-19 pandemic. In this study, we aimed to gain more insight into the relationship between caregiving and physical and mental health from the caregivers’ perspective in Serbia. Our results suggest that apart from providing care for a family member and the level of care, increased concerns about self-health and the health of the person being cared for due to the COVID-19 epidemic were predictors of the informal caregivers’ quality of life in Serbia.

European Union (EU) data demonstrate that 55%–60% of informal caregivers of the elderly are women, with ages from 50–59 years [14]. Similar data were obtained in our study, where the largest number of informal caregivers were females, mean age of 51 years. However, data from the United States [15] show that, although informal caregivers are mainly women, the number of men has recently risen to 40%. The increase in the number of male caregivers in the United States is likely due to a change in family structure and smaller numbers of children, which resulted in the decrease of potential informal caregivers available per family. Our study results did not indicate any discrepancies or gender differences in how burden is experienced.

In our study, the majority of informal caregivers currently provide care (81.2%) for a person who is a family member (92%) on a daily basis (91.1%) and live in the same household with the person they care for (76.8%). EU [16,17] data shows that almost 50% of the family caregivers are children of the elderly care recipients and that 20%–45% are partners/spouses. Furthermore, 48% live in the same household as the person they care for, while 18% live nearby. According to the US 2015 Caregiving Report [18], 44 million Americans over the age of 18 provide help and support to an elderly family member, friend, or adult with a disability. In our study, the median time spent in informal care was five years, which corresponds to data obtained in the EU.

Similar to the results of this study, numerous studies [19,20] have shown that there is a strong correlation between the level of care complexity and the subjective perception of caregivers’ health, where more demanding care corresponds to a worse perception of one’s own health. It should also be kept in mind that the level of care complexity was found to be among the main reasons influencing the decision to accommodate a functionally dependent person in a nursing home.

Providing care for a family member had a significant impact on physical and mental health in our study [21]. This finding may be related to social norms accepted by families, as well as family expectations of care intensity, but also to the personal perception of obligation and emotions felt by the informal caregiver (obligation, guilt, helplessness, etc.).

The results of research conducted in Hong Kong [3,22] in the early stages of the COVID-19 pandemic showed that a significant part (almost 25%) of the general population took the responsibility to provide informal care at home. Given the fact that they were most economically active, many had two significant causes of stress, the professional work they had to maintain and concurrently to provide informal care at home. In addition, many reported insufficient knowledge regarding care delivery and increased psychological stress.

The COVID-19 pandemic has globally reduced the informal caregivers’ abilities to provide optimal care to the care recipients on multiple levels. It should be kept in mind that elderly and persons with pre-existing health conditions have an increased risk of negative health outcomes related to COVID-19 than the general population [23]. Most of the undertaken public health efforts were aimed to prevent disease transmission among these high-risk social groups but also resulted in increased stress put on informal caregivers. Additional COVID-19 preventive measures, such as lockdowns, restricted access to healthcare services (especially for non-epidemic needs), and limited use of social protection services, place a lot of boundaries on a way of providing informal care. This has contributed to an increased effort informal caregivers need to provide. Moreover, the risk of the informal caregiver becoming infected has brought an additional level of stress, as being COVID-19 positive would mean instantaneous inability to provide care during self-isolation, regardless of the presence or absence of disease symptoms. Caregivers are experiencing new and stressful situations regarding the state of transmission in social isolation cases [24]. Many of the traumatic situations, such as loneliness and isolation, although unintended, prove to be hard for the caregiver and their patients in need [25,26]. They become more reluctant to ask for help, in fear of an outsider bringing the virus into their home. Although the solution could be found in the expansion of telehealth, special attention should be given to overcome the limited technological capacity of caregivers and care recipients in order to establish proper healthcare services delivery in the coming months. Worldwide, both in the short- and long-term, healthcare systems rely heavily on the healthcare delivery activities performed by informal caregivers [16,27,28]. However, due to demographic changes, aging populations, changes in family structure, as well as challenges related to professional commitments and financial boundaries, informal caregivers are becoming an increasingly limited resource; and this trend is prompt to become a continuum. An enduring challenge is to ensure that informal caregivers are supported by local communities and public health policies in order to preserve their health. Increased availability of social services, balanced coordination of formal and informal care, flexibility in working hours, formal and informal training can be of great value for supporting these irreplaceable but often forgotten healthcare workers during the COVID-19 emergency. Based on the results outlined in this study, we offer several recommendations that might be put in order to improve the health of informal caregivers during the COVID-19 pandemic. Results of this study are fully applicable to the population presented, and a certain number of them are possibly useful in a global sense. At the national and local level, decision-makers must ensure that formal and informal caregivers have appropriate protective equipment to be held safe from a potential infection. It is also necessary to receive appropriate training regarding the protection of themselves and those they care for. The training also ensures that caregivers receive clear protocols that will easily and understandably articulate techniques for protection and self-protection against the infection.

In regards to recognizing the role of informal caregivers as a part of a system of long-term care, it is necessary to develop protocols on clear communication between the formal and informal caregivers, including possible situations that are related to the outbreak of the epidemic. Planning potential transitional care is essential for promoting an adequate response. Ensuring that employers have an adequate attitude towards informal caregivers, especially during epidemics and emergencies, can contribute to reducing the deleterious effects on income and the general economic situation of caregivers.

It is necessary to have clear procedures and coordination of all systems during a state of emergency; responding to the needs of risk groups is possible only by recognizing the role of all sectors and institutions. This involves the inclusion of social protection systems and their protocols; if the needs for food, housing, and protective equipment are not met, they can trigger a number of negative outcomes for informal caregivers.

Institutions and organizations in the local community must act in solidarity and must take into account the needs that have informal caregivers. In some situations, they will need to provide assistance in procuring basic groceries, caring for children with disabilities, as well as more complex services such as microloans, or urgently accommodating a functionally dependent person if necessary. In Serbia, it turned out that there were volunteers who helped the elderly due to incapability of the elderly to go out, so they had the help of volunteers in procuring groceries or walks for children with disabilities.

The COVID-19 pandemic should be understood as a wake-up call to ensure adequate care for the elderly and people with disabilities. A strong public health system response in the form of urgent and joint action is needed to improve the preparedness and protection of at-risk groups. Public knowledge and awareness should be aimed at that scenarios where the caregivers are no longer able to physically be in the same place as the patient. In order to overcome these circumstances, developing telehealth tools and interventions would help to support informal caregivers. It should be recognized that not all caregivers and/or care recipients will have sufficient knowledge for this kind of communication, and additional guidance should be provided. Telemedicine is a new opportunity that should be adopted as a vital resource in the future, especially as the epidemic has shown that health monitoring and the provision of certain medical services at a distance can significantly contribute to improving health and quality of life.

## Figures and Tables

**Table 1:** Informal caregiver’s quality of life according to sociodemographic characteristics.

Variable	Physical Health		Mental Health	
	X ± SD	*p*	X ± SD	*p*
Sex		0.179		0.732
Male	59.6 ± 18.3		51.1 ± 21.6	
Female	52.7 ± 22.4		49.3 ± 22.8	
Age	−0.164 *	0.085	−0.108 *	0.258
Education		0.657		0.778
Elementary/Secondary	53.2 ± 24.1		50.3 ± 24.4	
College/University	55.1 ± 19.1		49.1 ± 20.6	
Retired		0.030		0.417
No	56.1 ± 21.6		50.4 ± 23.1	
Yes	44.0 ± 20.1		45.7 ± 19.1	
Community		0.479		0.296
Rural	58.2 ± 22.6		55.9 ± 24.1	
Urban	53.6 ± 21.7		48.9 ± 22.3	

X, mean; SD, standard deviation; * the correlation coefficient.

**Table 2:** Quality of life of informal caregivers according to care characteristics.

Variable	Physical Health		Mental Health	
	X ± SD	*p*	X ± SD	*p*
Care characteristics				
Caring for				
One person	53.5 ± 22.5	0.451	49.1 ± 23.1	0.592
More than one	58.3 ± 17.4		52.6 ± 19.4	
Live in a joint household with the person caring for				
No	57.5 ± 23.9	0.363	51.4 ± 24.8	0.663
Yes	53.1 ± 21.1		49.2 ± 21.9	
Caring for a family member				
No	76.7 ± 16.3	0.001	71.9 ± 18.6	0.002
Yes	52.2 ± 21.1		47.8 ± 21.8	
Providing care frequency				
Daily	61.9 ± 17.1	0.237	59.4 ± 18.0	0.153
Less than daily	53.4 ± 22.1		48.7 ± 22.7	
Someone else participates and helps in providing care				
No	51.7 ± 20.7	0.242	47.8 ± 21.3	0.369
Yes	56.5 ± 22.7		51.6 ± 23.6	
Level of care complexity	−0.319 *	0.001	−0.336 *	<0.001
Duration of care	−0.189 *	0.062	−0.122 *	0.230
COVID-19 pandemic related questions				
Do you think that your health is more endangered now compared to the period before the coronavirus pandemic?				
No	57.3 ± 21.4	0.048	52.0 ± 23.2	0.169
Yes	49.0 ± 21.5		46.0 ± 21.2	
Do you now fear more for yourself and the health of the person you are caring for?				
No	60.3 ± 22.4	0.022	57.5 ± 23.8	0.006
Yes	50.5 ± 20.7		45.2 ± 20.7	

X, mean; SD, standard deviation; * Correlation coefficient.

**Table 3:** Univariate and multiple regression analysis for physical and mental health domains.

Variable	Univariate Analysis			Multivariate Analysis		
	B	SE	*p*	B	SE	*p*
**Physical Health**						
Retired vs. other categories of employment	−12.1	5.5	0.030			
Caring for family member	−24.5	7.2	0.001	−21.5	6.7	0.002
Level of care complexity (0–10)	−2.7	0.8	0.001	−2.9	0.8	<0.001
Belief that caregivers health is more endangered than before the pandemic	−8.3	4.1	0.048			
Increased concern about self-health and the health of the person being cared for	−9.9	4.2	0.022			
**Mental health**						
Caring for family member	−24.1	7.5	0.002			
Level of care complexity (0–10)	−3.0	0.8	<0.001	−2.8	0.8	<0.001
Increased concern about self-health and the health of the person being cared for	−12.3	4.3	0.006	−21.8	7.2	0.003

B, regression coefficient; SE, standard error.
